# Light‐Controllable Binary Switch Activation of CAR T Cells

**DOI:** 10.1002/cmdc.202100722

**Published:** 2022-02-21

**Authors:** Aya Kobayashi, Alberto Nobili, Steven C. Neier, Amissi Sadiki, Robert Distel, Zhaohui Sunny Zhou, Carl D. Novina

**Affiliations:** ^1^ Department of Cancer Immunology and Virology Dana-Farber Cancer Institute Boston MA 02215 USA; ^2^ Department of Medicine Harvard Medical School Boston MA 02115 USA; ^3^ Broad Institute of Harvard and MIT Cambridge MA 02142 USA; ^4^ Department of Chemistry and Chemical Biology Northeastern University Boston MA 02115 USA; ^5^ Barnett Institute of Chemical and Biological Analysis Northeastern University Boston MA 02115 USA; ^6^ Dynamic Cell Therapies, Inc. 127 Western Ave. Allston MA 02134 USA; ^7^ Binney Street Capital Boston MA 02215 USA

**Keywords:** Immunology, Cage compounds, Cancer, Photolysis, Fluorescence

## Abstract

Major challenges to chimeric antigen receptor (CAR) T cell therapies include uncontrolled immune activity, off‐tumor toxicities and tumor heterogeneity. To overcome these challenges, we engineered CARs directed against small molecules. By conjugating the same small molecule to distinct tumor‐targeting antibodies, we show that small molecule specific‐CAR T cells can be redirected to different tumor antigens. Such binary switches allow control over the degree of CAR T cell activity and enables simultaneous targeting of multiple tumor‐associated antigens. We also demonstrate that ultraviolet light‐sensitive caging of small molecules blocks CAR T cell activation. Exposure to ultraviolet light, uncaged small molecules and restored CAR T cell‐mediated killing. Together, our data demonstrate that a light‐sensitive caging system enables an additional level of control over tumor cell killing, which could improve the therapeutic index of CAR T cell therapies.

## Graphical abstract

**UV license to kill**: Ultraviolet‐light‐sensitive caged small molecules are coupled to tumor‐targeting antibodies, which work as an on/off switch for small‐molecule‐specific chimeric antigen receptor (CAR) T cells. Upon exposure to ultraviolet light, small molecules are uncaged and CAR T‐cell‐mediated killing of tumor cells is restored. This biorthogonal uncaging strategy could enable the temporal and spatial control over tumor cell killing of the CAR T cells.

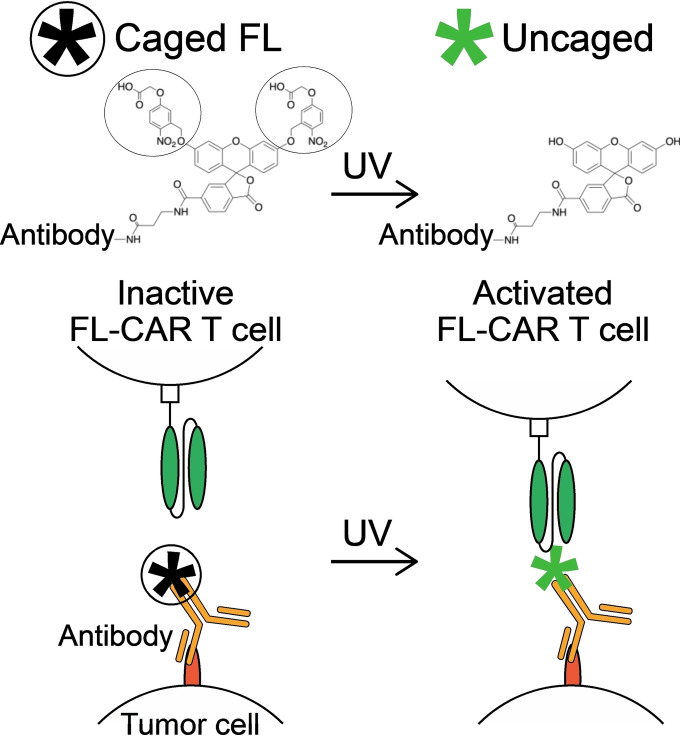

The adoptive transfer of T cells expressing chimeric antigen receptors (CARs) have demonstrated remarkable success against B cell malignancies; however, the efficacy of CAR T cells is limited to certain subsets of leukemias and has not shown a clinical benefit for patients with solid tumors.^[1]^ Most solid tumors do not express a unique surface antigen that is also not present on tissues required for viability. Even low‐level expression of a protein on the surface of healthy tissue can cause ‘on‐target’ but ‘off‐tumor’ toxicities, in which healthy tissues expressing the same antigen as tumor cells are targeted by CAR T cells. These issues have limited the development of CAR T cells against solid tumors.^[2]^

Certain CAR T cells targeting EGFR,^[3]^ EGFRvIII,^[4]^ IL13Rα2,^[5]^ mesothelin,^[6]^ carcinoembryonic antigen (CEA),^[7]^ and prostate‐specific membrane antigen (PSMA)^[8]^ have demonstrated reasonable safety but limited efficacy against solid tumors. Solid tumors are often heterogenous with sub‐populations of tumor cells expressing different antigens, which contributes to the limited clinical benefit of CAR T cell therapies. As a result, there is also critical need for technologies that can redirect CAR T cells against multiple antigens, which can improve CAR T cell efficacy against solid tumors.

Strategies to improve CAR T cell specificity include a synthetic Notch receptor (synNotch) CAR (Boolean AND gate logic)^[9]^ and a split, universal, and programmable (SUPRA) CAR (Boolean AND‐NOT gate logic).^[10]^ These CARs become fully functional upon binding to antigens on the surface of tumors. These systems can enhance the specificity of CAR T cells but can be limited to tumors expressing only specific combinations of antigens, which increases the risk of tumor antigen escape.

To enhance safety and efficacy, doseable CAR T cell systems have been developed. In these systems, CARs become active when a doseable agent is administered. Previously described doseable CARs include SUPRA CAR,^[10]^ biotin‐CAR,^[11]^ 5B9 tag‐CAR (UniCAR),^[12]^ GCN4 peptide‐CAR^[13]^ and fluorescein (FL) CARs.[^13a^, ^14^] By conjugating tags or peptides to tumor‐targeting antibodies of distinct antigen specificities, one universal CAR T cell can be redirected to target multiple antigens on the surface of cancer cells. Of the doseable CAR T cell systems, we favor small molecule CARs such as FL CARs. Small molecules are often non‐immunogenic, easy to modify and manufacture, and fluorescein has been used clinically in angiography without severe side effects.^[15]^

To address the limitations of current CAR T cell therapies, here we present a strategy that allows for a universal CAR T cell to control the timing and degree of activation against multiple antigens. Conventional CAR T cells with fixed antigen specificity intrinsically couple tumor binding with tumor killing. We engineered a small molecule‐specific CAR, which uncouples tumor binding from tumor killing. Small molecule‐specific CARs bind to a small molecule coupled to an antibody, which binds to tumor‐associated antigens. The binding of the antibody‐small molecule conjugate functions as a binary switch (Figure 1A), thereby allowing controlled CAR T cell activity. The binary event is binding of antibody‐small molecule conjugates to the surface of target cells. The activity of this CAR T cell is entirely dependent on the presence of the antibody‐small molecule conjugate.

**Figure 1:**
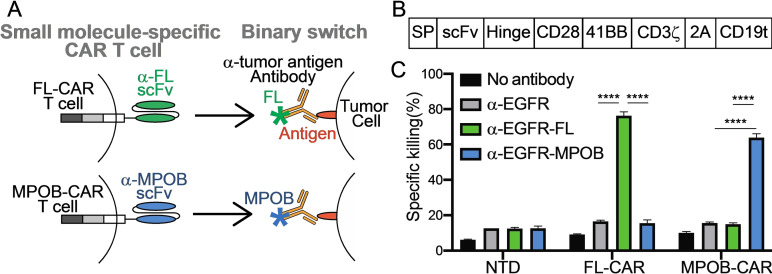
**Small molecule‐specific CAR T cell killing** (A) Schematic representation of the small molecule specific‐CAR T cells against fluorescein (FL) (Upper) and MPOB (Lower). (B) Diagram showing the construction of anti‐small molecule CAR, where SP; signal peptide, scFv; single chain variable fragment that recognizes small molecules, Hinge; hinge domain from CD8, CD28; transmembrane and costimulatory domains from CD28, 4‐1BB; costimulatory domain from CD137, CD3ζ; activation domain from CD3ζ; 2 A: 2 A peptide, CD19 t (truncated CD19); extracellular and transmembrane domains from CD19 (C) MDA‐MB‐468 cells were labeled with α‐EGFR‐FL or α‐EGFR‐MPOB, and antigen‐specific cytotoxicity was tested after co‐incubation of labeled MDA‐MB‐468 cells with non‐transduced (NTD), FL‐CAR and MPOB‐CAR T cells. (n=3). *P* values were determined by unpaired Student's *t* test. ****P<0.0001.

Each small molecule specific‐CAR is composed of a small molecule specific single‐chain variable fragment (scFv) followed by intracellular signaling domains (Figure 1A). For these studies, we used two different scFvs specific to fluorescein (FL) or 4‐[(6‐methylpyrazin‐2‐yl)oxy]benzoic acid (MPOB) incorporated into a CAR construct consisting of the human CD8 hinge, CD28 transmembrane, CD28 and 4‐1BB costimulatory and CD3ζ activation domains (Figure 1B). FL‐CAR and MPOB‐CAR constructs were transduced into human primary T cells using retroviruses (Figure S1). We also generated two binary switches using anti‐EGFR antibody conjugated with fluorescein (α‐EGFR‐FL) or MPOB (α‐EGFR‐MPOB).

To assess the small molecule specificity of our CAR T cell system, MDA‐MB‐468 cells expressing EGFR were labeled with α‐EGFR‐FL or α‐EGFR‐MPOB, and then co‐cultured with FL‐ or MPOB‐specific CAR T cells *in vitro*. FL‐ and MOPB‐CAR T cells demonstrated small molecule specific cytotoxicity because the FL‐CAR did not kill cells coated with antibody‐MPOB and the MPOB‐CAR did not kill cells coated with antibody‐FL (Figure 1C).

Next, we compared the cytotoxicity of small molecule‐specific CAR T cells with that of conventional anti‐EGFR CAR T cells. The 1YY9^[16]^ scFv was used to generate anti‐EGFR‐CAR T cells. FL‐CAR T cells showed comparable cytotoxicity to conventional anti‐EGFR‐CAR T cells (Figure S2). FL‐CAR T cells had background cytotoxicity against unlabeled MDA‐MB‐468 cells but showed antigen specific cytotoxicity against MDA‐MB‐468 cells labeled with α‐EGFR‐FL (Figure S2). Consistent with cytotoxicity data, co‐culture of FL‐CAR T cells with α‐EGFR‐FL antibody and MDA‐MB‐468 cells led to increased expression of activation markers, IL‐2 and IFN‐γ by FL‐CAR T cells (Figure S3). Co‐culture of FL‐CAR T cells without antibody or with an unlabeled α‐EGFR antibody did not affect FL‐CAR T cell activation or cytotoxicity.

CAR T cell activation is associated with a higher density of antigens on target cells.^[17]^ To test whether increasing the density of small molecule antigens on tumor‐targeting antibodies affects FL‐CAR T cell activation, we conjugated anti‐EGFR antibodies with increasing amounts of fluorescein (Figure S4–S6, Table S1). FL‐CAR T cells showed weak cytotoxicity with low fluorescein density on antibodies (fluorescein to antibody ratio 1.4), while the cytotoxicity increased 3.5‐fold and 9.8‐fold with a fluorescein to antibody ratio of 2.4 and 11, respectively (Figure S7). These data indicate that increasing fluorescein density per antibody increases FL‐CAR T cell cytotoxicity against targeted tumor cells. Therefore, CAR T cell activity may be titrated by the density of small molecules on the tumor‐targeting antibodies independently of the density of antigen on the surface of tumor cells.

In addition to modulating small molecule density on tumor‐targeting antibodies, titrating antibody‐small molecule conjugates can control the activity of small molecule specific‐CAR T cells. To assess titratable CAR T cell activity, MDA‐MB‐468 cells were labeled with increasing doses of α‐EGFR‐FL at a fluorescein to antibody ratio of 11, and co‐cultured with FL‐CAR T cells. These data demonstrate that the cytotoxicity of FL‐CAR T cells increased in dose‐dependent manner (Figure 2).

**Figure 2:**
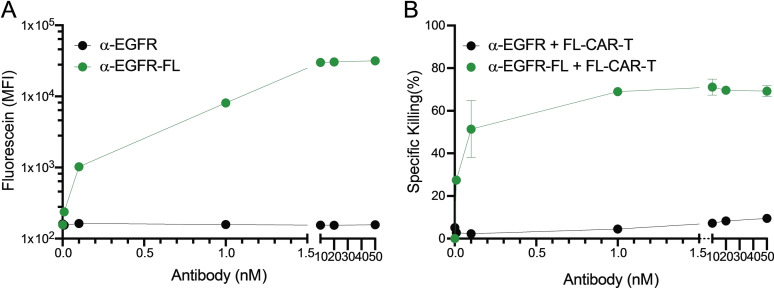
**Small molecule‐dose and density‐dependent activation of CAR T cell** (A) MDA‐MB‐468 cells were labeled with different concentrations of α‐EGFR or α‐EGFR‐FL (fluorescein to antibody ratio=11) and the fluorescence intensity was measured by flow cytometry (n=3). (B) Cytotoxicity of FL‐CAR T cells were tested after co‐culture with these labeled MDA‐MB‐468 cells (n=3).

To assess FL‐CAR T cell activation by fluorescein in the absence of tumor cells, FL‐CAR T cells were cultured on plates coated with increasing doses of antibody‐fluorescein conjugates (Ab‐FL) or cultured with free floating Ab‐FL (Figure S8). Consistent with the activation by fluorescein on tumor cells, plate‐bound‐fluorescein successfully activated FL‐CAR T cells and increased expression of IL‐2 and IFN‐γ in CAR T cells in a fluorescein‐dose‐dependent manner (Figure S8). Free floating Ab‐FL failed to activate FL‐CAR T cells as efficiently as plate‐bound Ab‐FL. These results demonstrate titratable CAR T cell activity based on the density of fluorescein on antibodies and suggest that optimizing the density of small molecules on the antibody and the dose of the antibody‐small molecule conjugates might lead to a wider therapeutic index for small molecule specific‐CAR T cells.

To evaluate simultaneous targeting of multiple tumor antigens, T cells expressing FL‐CAR were used to target EGFR, HER2, and CD38 expressed on breast cancer (MDA‐MB‐468 and HCC1954), ovarian cancer (OVCAR8), Burkitt Lymphoma (Ramos and Raji), and multiple myeloma (H929) cells (Figure 3A and Figure S9). Each cancer cell line was labeled with fluorescein‐conjugated antibodies against EGFR (α‐EGFR‐FL), HER2 (α‐HER2‐FL), CD38 (α‐CD38‐FL) and were cocultured with FL‐CAR T cells. As expected, cytotoxicity of FL‐CAR T cells was specific to the antibody‐fluorescein conjugates for all targets and cell lines tested (Figure 3A and Figure S10). These results suggest that FL‐CAR T cells can be redirected to a variety of targets on different types of tumors including solid tumors by using fluorescein‐conjugated antibodies with distinct antigen specificity.

**Figure 3:**
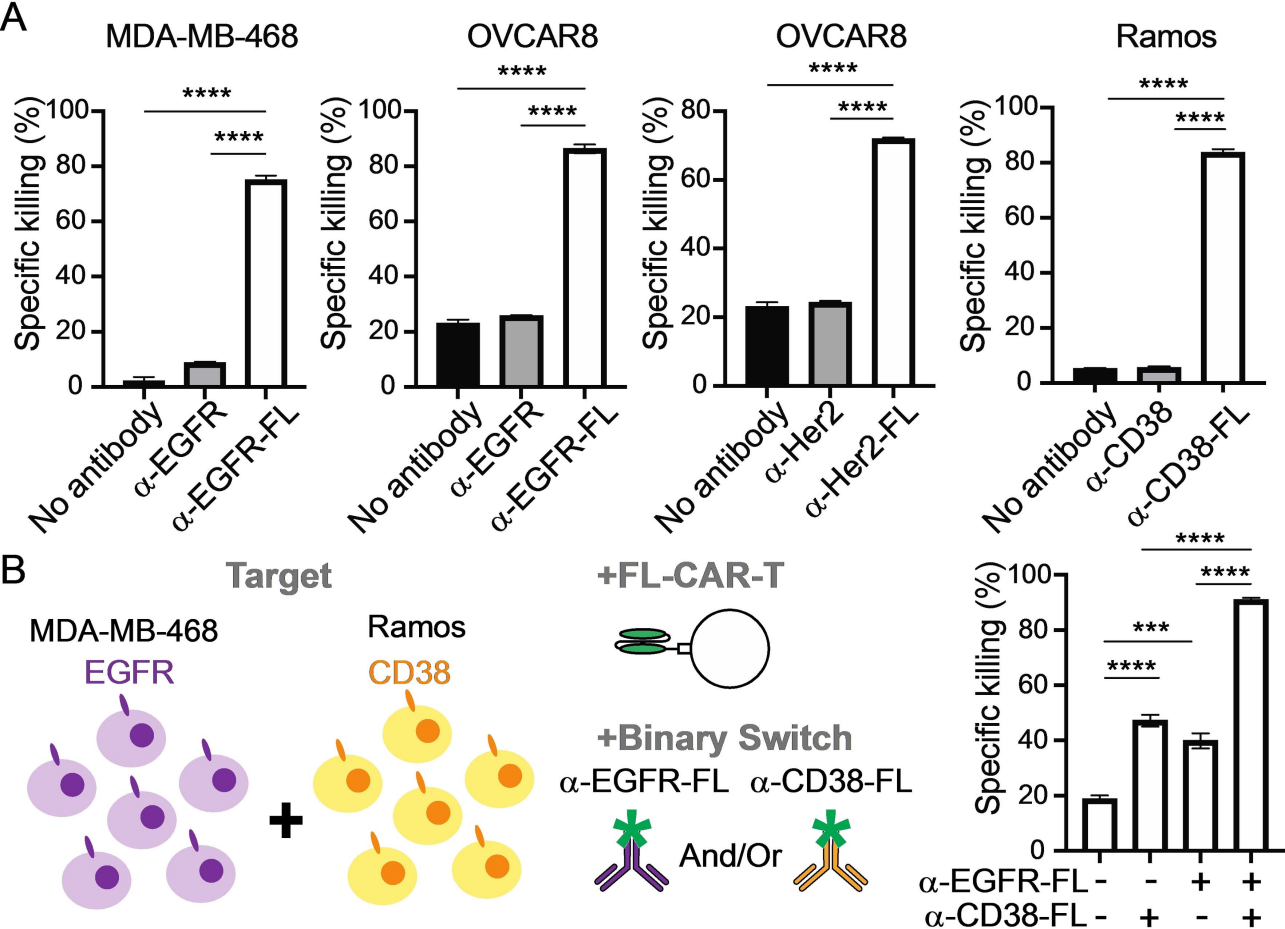
**Dual targeting by a redirectable small molecule specific‐CAR** (A) Cancer cell lines were labeled with naked or fluorescein‐conjugated antibodies specific to each antigens, and cytotoxicity of FL‐CAR T cells were tested after co‐culture with these labeled cancer cell lines (n=3). (B) MDA‐MB‐468 (EGFR+) and Ramos (CD38+) cells were mixed at 1 : 1 ratio and labeled with α‐EGFR‐FL and/or α‐CD38‐FL, and cytotoxicity of FL‐CAR T cells were tested after co‐culture with this mixture of cancer cell lines (n=3). *P* values were determined by unpaired Student's *t* test. **P<0.01, ***P<0.001, ****P<0.0001.

To mimic CAR T cell killing of heterogeneous cell populations, we mixed EGFR‐expressing MDA‐MB‐468 cells and CD38‐expressing Ramos cells at 1 : 1 ratio and labeled this mixed cell population with labeled α‐EGFR‐FL alone, α‐CD38‐FL alone or cocktail of α‐EGFR‐FL and α‐CD38‐FL (Figure 3B and Figure S11). FL‐CAR T cells killed only the EGFR+ MDA‐MB‐468 cells when tumor cell mixture was treated with α‐EGFR‐FL, whereas the EGFR‐ Ramos cells were not killed by FL‐CAR T cells. Similarly, FL‐CAR T cells killed only the CD38+ Ramos cells when labeled with α‐CD38‐FL, whereas CD38‐ MDA‐MB‐468 cells were not killed by FL‐CAR T cells. Combining α‐EGFR‐FL and α‐CD38‐FL led to FL‐CAR T cell‐mediated killing of both MDA‐MB‐468 cells and Ramos cells. These results demonstrate that single specificity FL‐CAR T cells can simultaneously target multiple tumor antigens and kill heterogenous cell populations.

To add an additional layer of control over CAR T cell activity, we integrated photocaging technologies and small molecule coupling chemistries with CAR T cell biology. We coupled an ultraviolet (UV)‐light sensitive ‘cage’ on fluorescein to tumor‐targeting antibodies. Covalently adding two 5‐carboxymethoxy‐2‐nitrobenzyl (CMNB) caging groups is expected to block FL‐CAR T cell binding to fluorescein molecules and prevent FL‐CAR T cell killing (Figure 4A). Conversely, UV‐light irradiation is expected to dissociate the CMNB‐caging groups, which exposes fluorescein and permits FL‐CAR T cell killing.

**Figure 4:**
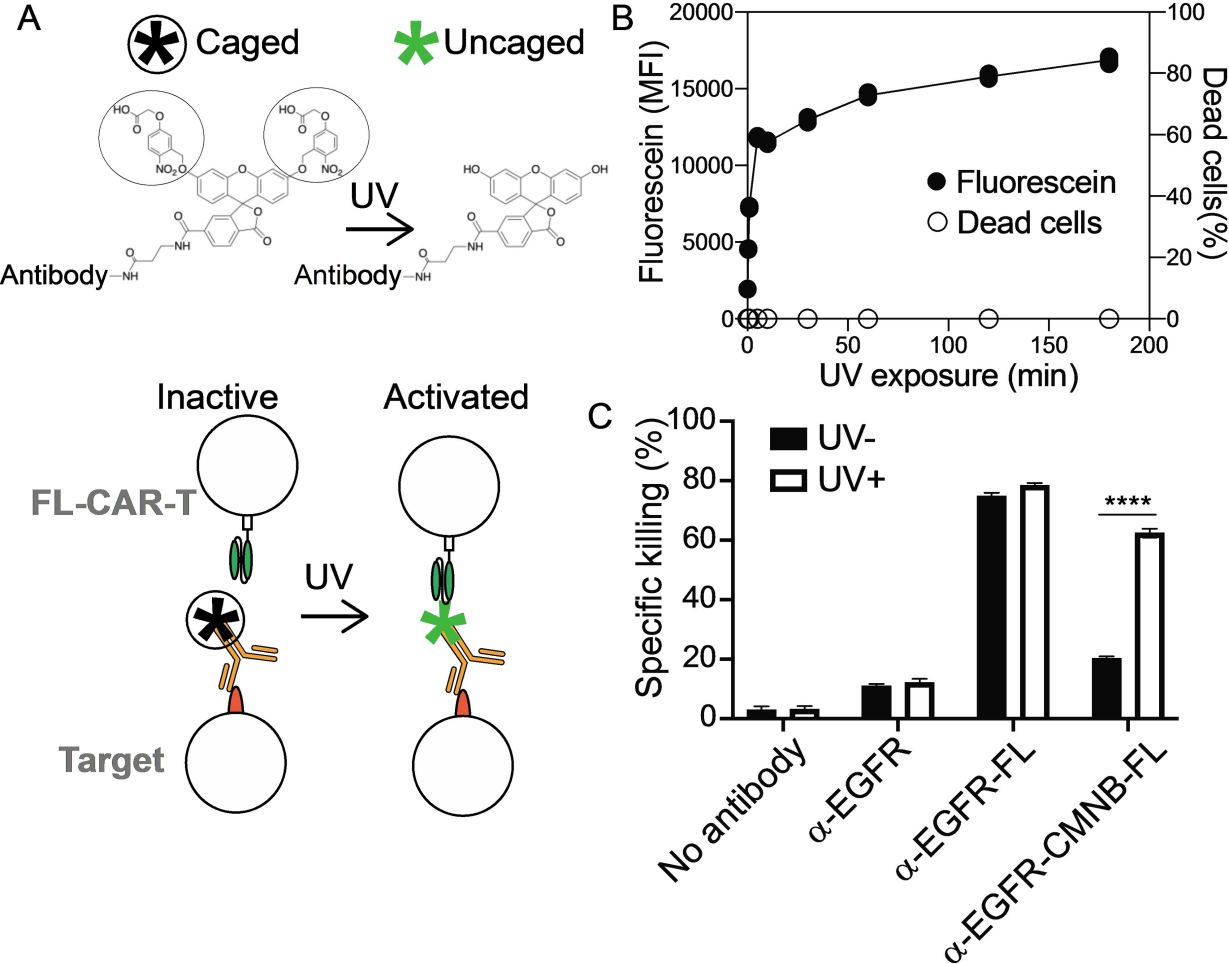
**UV light‐sensitive caging blocks small molecule‐mediated CAR T cell killing** (A) Schematic representation of the UV light‐sensitive caging strategy. CMNB‐caging groups protect fluorescein from FL‐CAR T cell recognition. CMNB‐caged fluorescein becomes recognizable by the FL‐CAR T cell after UV exposure and removal of the CMNB‐caging groups. (B) MDA‐MB‐468 cells were labeled with α‐EGFR‐CMNB‐FL and then irradiated with UV light for up to 3 hr, and the fluorescence intensity (solid circle) and cell death (open circle) was measured (n=3). (C) MDA‐MB‐468 cells were labeled with α‐EGFR, α‐EGFR‐FL or α‐EGFR‐CMNB‐FL and treated with or without UV‐light for 10 min. Cytotoxicity of FL‐CAR T cells were tested after co‐culture with these cancer cells (n=3). *P* values were determined by unpaired Student's *t* test. ****P<0.0001.

To test whether UV‐light sensitive caging can control tumor cell killing, anti‐EGFR antibodies were conjugated with the CMNB‐caged fluorescein (α‐EGFR‐CMNB‐FL). MDA‐MB‐468 cells were labeled with α‐EGFR‐CMNB‐FL then exposed to 365 nm UV‐light for up to 3 hours. Our data demonstrate that UV‐light exposure leads to removal of CMNB‐cages with near maximal fluorescence intensity detected after 10 minutes of UV‐light exposure (Figure 4B). Cellular damage caused by UV‐light exposure was negligible.

MDA‐MB‐468 cells labeled with α‐EGFR‐FL (uncaged control) were susceptible to FL‐CAR T cell killing, whereas cells labelled with α‐EGFR‐CMNB‐FL were resistant to FL‐CAR T cell killing. UV‐light exposure induced killing of tumor cells labelled with α‐EGFR‐CMNB‐FL comparable to the level of killing of cells labeled with α‐EGFR‐FL (Figure 4C and Figure S12). Consistent with this observation, FL‐CAR T cell activation as assessed by CD69 and cytokine production as assessed by IL‐2 and IFN‐γ were comparable between cells labeled with α‐EGFR‐FL cells labelled with α‐EGFR‐CMNB‐FL after exposure to UV‐light (Figure S12). These results demonstrate that CAR T cell killing can be controlled by photocaging the small molecule when conjugated to antibodies.

One possible concern for the clinical adoption of this technology is poor tissue penetrance of UV light. UV light might be used in dermal applications and in dermabrasion for cutaneous applications of this technology. For subdermal applications, it is possible to use tools such as optical lens‐microneedle array^[18]^ and UV light emitting diode (LED) coupled to an optical fiber^[19]^ that would enable spatiotemporal activation of CAR T cells. The optical window for photomedicine applications is between 650 to 1300 nm.^[20]^ Photocaging groups such as BODIPY or cyanine that are uncaged at longer wavelengths up to 700 nm (near infrared) region have been described^[21]^ and can be incorporated. Moreover, therapeutic optogenetics using 590 nm are currently in development.^[22]^

Here we present a novel strategy that couples small molecule activation of CAR T cells with small molecule photocaging. The stability of the photocage and the wavelength of light used for destabilizing the photocage determine the area and time that CAR T cells would react at accessible tissues. The choice of advanced light‐delivering technologies would determine which tissues might be targetable by light‐controlled CAR T cells. One can imagine the use of light‐controlled regulatory CAR T (CAR Treg) cells for organ transplantation where cell surface proteins such as the human leukocyte antigen (HLA) on donor organs but unshared by the recipient can be targeted.^[23]^ Light‐controlled CAR Treg therapy can also be considered for autoimmune diseases such as type 1 diabetes and rheumatoid arthritis,[^23a^, ^24^] in which appropriate antigen targets have been identified. The strategies described here will accelerate adoption of spatiotemporal control of CAR T cell therapies in clinical applications.

## Conflict of interest

A.N., S.C.N., R.D., and C.D.N. filed patents on the small molecule CAR and caging technologies described in this manuscript. A.N., R.D., and C.D.N. and have equity in Dynamic Cell Therapies, Inc. S.C.N., R.D., and C.D.N. are consultants to Dynamic Cell Therapies, Inc. A.N. is currently employed by Dynamic Cell Therapies, Inc.

## Supporting information

As a service to our authors and readers, this journal provides supporting information supplied by the authors. Such materials are peer reviewed and may be re‐organized for online delivery, but are not copy‐edited or typeset. Technical support issues arising from supporting information (other than missing files) should be addressed to the authors.

Supporting Information

## Data Availability

Data sharing is not applicable to this article as no new data were created or analyzed in this study.
