# Supplementary material for: Light‐Controllable Binary Switch Activation of CAR T Cells
Source: ChemMedChem. 2022 Feb 21;17(12):e202100722. doi: 10.1002/cmdc.202100722 (PMC9304291; doi:10.1002/cmdc.202100722)
Supplement: Supplementary file 1 — Supporting Information [file CMDC-17-0-s001.pdf]

ChemMedChem

Supporting Information

Light-Controllable Binary Switch Activation of CAR T Cells

Aya Kobayashi[+], Alberto Nobili[+], Steven C. Neier, Amissi Sadiki, Robert Distel, Zhaohui Sunny Zhou, and Carl D. Novina*

Table of Contents

1. Supplementary Methods
2. Supplementary Figures
3. Supplementary Table
4. Author Contributions
5. Supplementary References

1. Supplementary Methods

Cell lines, media and reagents

Following cell lines were cultured in DMEM (Life Technologies): PG13 (ATCC, Cat# CRL-10686) and Platinum-E (Cell Biolabs, Cat# RV-101).

Following cell lines were cultured in RPMI-1640 (Life Technologies): MDA-MB-468 (ATCC, Cat# HTB-132), HCC1954 (ATCC, Cat# CRL-2338, RRID:CVCL_1259), Raji (ATCC, Cat# CCL-86), OVCAR8 (a generous gift from Dr. Sarah L. Hill) and H929 (a generous gift from Dr. Nikhil C. Munshi).

DMEM and RPMI-1640 media were supplemented with 10% FBS.

Cells were tested for Mycoplasma contamination with a commercial detection kit (MycoAlert, Lonza, Cat# LT07-118).

Human PBMCs from healthy donors were obtained from Brigham and Women's Hospital under a Dana-Farber Cancer Institute (DFCI) Institutional Review Board (IRB)-approved protocol (Protocol No. 18-564). PBMCs were obtained by Ficoll-isolation (GE Healthcare) and T cells were isolated by EasySep Human T Cell Isolation Kit (Stemcell Technologies, Cat# 17951). T cells were cultured in X-vivo 15 (Lonza, Cat# 30661) supplemented with 5% human AB serum (Gemini Bioproducts, Cat# 100-512), 1% Gluta-Max (ThermoFisher Scientific) and recombinant human IL-2 (Peprotech, Cat# 200-02).

CAR construction

The anti-FL-CAR, anti-MPOB-CAR and anti-EGFR-CAR were generated by fusing the signal peptide of CD8 to the anti-fluorescein scFv antibody 4M5.3[1] or anti-MPOB scFv (Randox) or anti-EGFR scFv 1YY9[2], followed by CD8 hinge, CD28 transmembrane, CD3ζ and 4-1BB cytoplasmic, and CD19 extracellular and transmembrane (truncated CD19) coding sequences. Truncated CD19 was used as a reporter gene.

These sequences were cloned into the Moloney murine leukemia virus-based gamma-retroviral vector pSFG[3].

Retrovirus production and transduction

Platinum-E (Plat-E) retroviral packaging cells (Cell Biolabs) were transiently transfected with retroviral plasmids encoding the CARs via Lipofectamine 2000 (Life Technologies). Supernatants were collected 48 h after transfection. PG13 producer cells (ATCC) were then transduced with supernatants, and the viral supernatants produced from transduced PG13 cells were obtained.

Human primary T cells were activated with anti-CD3/CD28 beads (Life Technologies) with recombinant human IL-2 (Peprotech). Activated T cells were retrovirally transduced with CAR retrovirus supernatant in Retronectin (Takara)-coated plates. CAR expression was confirmed by flow cytometry using truncated CD19 marker.

Conjugation of small molecules to antibodies

Detailed method is described in supplemental information. Briefly, fluorescein was conjugated to anti-EGFR antibody (Cetuximab), anti-HER2 antibody (Pertuzumab), and anti-CD38 antibody (Daratumumab) by using NHS-Fluorescein Antibody Labeling Kit (ThermoFisher Scientific). For the conjugation of CMNB-caged fluorescein, CMNB-Caged Carboxyfluorescein, SE (5-Carboxyfluorescein-Bis-(5-Carboxymethoxy-2-Nitrogenzyl Ether, beta-Alanine-Carboxamide, Succinimidyl Ester (ThermoFisher Scientific) was used. For MOPB conjugation, 4-[(6-Methylpyrazin-2-yl)oxy]benzoic acid (FisherScientific) was conjugated to anti-EGFR antibody by using Amine Coupling Kit (Dojindo).

Cytotoxicity, activation and cytokine assays.

Target tumor cells were labeled with CellTrace dye violet or yellow (ThermoFisher Scientific) and TAA-specific antibodies conjugated with small molecules, and then 100,000 tumor cells were co-incubated with CAR T cells for 12-19 h at effector-to-target (E:T) ratio = 1:1. For unmasking of CMNB-caging group, cells were irradiated with 365nm UV light at 300 $\mu W/m^2$ for indicated time periods. Cells were collected and stained with Zombie NIR Fixable Viability dye and evaluated by flow cytometry analyses. Specific killing was calculated as (experimental death – spontaneous death)/(100 – spontaneous death) X 100%. Activation of CAR T cells was evaluated by co-incubation with target cells at 1:1 ratio for 5 h. Cytokine production by CAR T cells was evaluated by co-incubation with target tumor cells at 1:1 ratio for 12 h.

Flow cytometry analysis

All samples were analyzed with an LSR Fortessa (BD Bioscience) and data were analyzed using FlowJo software (FlowJo, RRID: SCR_008520). Truncated CD19 was detected with anti-CD19 antibody (clone HIB19, Biolegend). T cell phenotype was evaluated with anti-CD4 antibody (clone OKT4, Biolegend, Cat# 317442), anti-CD8 antibody (clone HIT8a, Biolegend, Cat# 300934), anti-CD69 antibody (clone FN50, Biolegend, Cat# 310910), anti-IL-2 antibody (clone MQ1-17H12, Biolegend, Cat# 500326), anti-IFN-γ antibody (clone 4S.B3, Biolegend, Cat# 502526).

Statistics. Data presented as means ± SD. Results were analyzed by Student's t test, and statistical significance was calculated using a Student's *t*-test. $P < 0.05$ was considered significant and is designated with an asterisk in figures. Statistical analyses were done with Prism software (GraphPad Prism, RRID:SCR_002798).

Site-specific Conjugation via Glycan Remodeling

The concentrations of the peptides and proteins were determined using UV absorption at 280 nm and extinction coefficients based on the antibody's amino acid sequence. All aqueous solutions were prepared using Milli-Q water.

The reactions were performed as previously described[4] based on manufacturer's recommendation (Genovis, L1-AZ1-200). First, the deglycosylation reaction contained 20 mM tris-buffered saline (TBS, pH 7.4), immobilized endoglycosidase (EndoS2, E.C. 3.2.1.96, GlycINATOR), 26 μM cetuximab and incubated at 23°C for 4 h. To remove excess unreacted reagents, each reaction mixture was desalted using 30 kDa MWCO centrifugal filters (Amicon, UFC503096) into 25 mM TBS (pH 7.4). Second, the azide installation reaction contained 20 mM TBS (pH 7.4), buffer additive, UDP-N-azidoacetylgalactosamine (UDP-GalNAz), β-1,4-galactosyltransferase (Y289L) (GalT, E.C. 2.4.1.38), 11 μM cetuximab and incubated at 30°C for 20 h and 30 min. To remove excess unreacted reagents, each reaction mixture was desalted using 50 kDa MWCO centrifugal filters (Amicon, UFC505096) into 25 mM TBS (pH 7.4). Third and lastly, the strain-promoted azide-alkyne cycloaddition (SPAAC) click reaction contained 20 mM TBS (pH 7.4), 345 μM dibenzocyclooctyne fluorescein (DBCO-FITC, compound 2, Fig S1, Lumiprobe, 15120), 6.9 μM cetuximab and incubated at 37°C for 20 h and 45 min. To remove excess unreacted reagents, each reaction mixture was desalted using 30 kDa MWCO centrifugal filters (Amicon unit, UFC503096) into 25 mM TBS (pH 7.4)

Amine-reactive Acylation Reaction

The reaction contained 100 mM potassium phosphate (pH 7.2), 138 μM N-hydroxysuccinimide fluorescein (NHS-FITC, compound 1, Fig S1, Lumiprobe, 151F0), 6.8 μM cetuximab (without deglycosylation), and incubated at 23°C. Aliquots (127 mL) of the reaction mixture were removed at different times (30 min, 1 h and 18 h), and then each aliquot was quenched by 2 mM Tris (pH 8). To remove excess unreacted reagents, each reaction mixture was desalted using 30 kDa MWCO centrifugal filters (Amicon unit, UFC503096) into 100 mM potassium phosphate (pH 7.6).

IdeS-mediated Proteolysis of Cetuximab

Modified and native cetuximab (final concentration: 0.2 g/l or 1 mM) in 25 mM TBS (pH 7.4) was digested by IdeS (Genovis, FabRICATOR, A0-FR1-020, E.C. 3.4.22.10) at 1 unit of IdeS protease per mg of antibody at 37°C for 5 h. SDS-PAGE analysis was performed as described in the next section.

Determination of fluorescein-antibody ratio

The loading ratio was calculated based on the ratio of the absorption of fluorescein and antibody, as previously described in the literature. Briefly, the absorbance spectra of antibody-fluorescein conjugates were collected from 220 to 750 nm (NanoDrop spectrophotometer, ND-1000). The concentration of the chromophore fluorescein was calculated using the absorbance of the conjugates at 494 nm and extinction coefficient of 75,000 $M^{-1}cm^{-1}$, provided by Lumiprobe. The antibody concentration was estimated by subtracting fluorescein's contribution at 280 nm and the calculated extinction coefficient based on its amino acid sequence (217,440 $M^{-1}cm^{-1}$ for Cetuximab). A summary of each conjugate's ratio is provided in Table S1.

Characterization of the Conjugates by SDS-PAGE

Sodium dodecyl sulfate–polyacrylamide gel electrophoresis (SDS-PAGE) was performed using a Bio-Rad Mini-PROTEAN 3 system or Criterion Cell. First, the reaction mixture was incubated with SDS Sample Buffer at 80 °C

for 10 min. For reducing and nonreducing gels, 4X reducing SDS sample buffer (Boston Bio Products, BP-110R) and 2X nonreducing SDS sample buffer (Bio- Rad, 1610737) were used, respectively. Second, the samples were loaded into 12% tris-tricine precast protein gels (Bio-Rad, 4561044) or 4–15% tris-glycine precast protein gels (Biorad, 5671083). Precision Plus Protein™ Dual Xtra Prestained Protein Standards (Bio-Rad, 1610377) were used for mass calibration. Electrophoresis was then performed at 200 V for 20 min. The gel was stained by Coomassie R250 and then destained using 10% acetic acid and 40% methanol. The gels were imaged using an iBright FL1000 Imaging system (Thermo Fisher Scientific).

2. Supplementary Figures

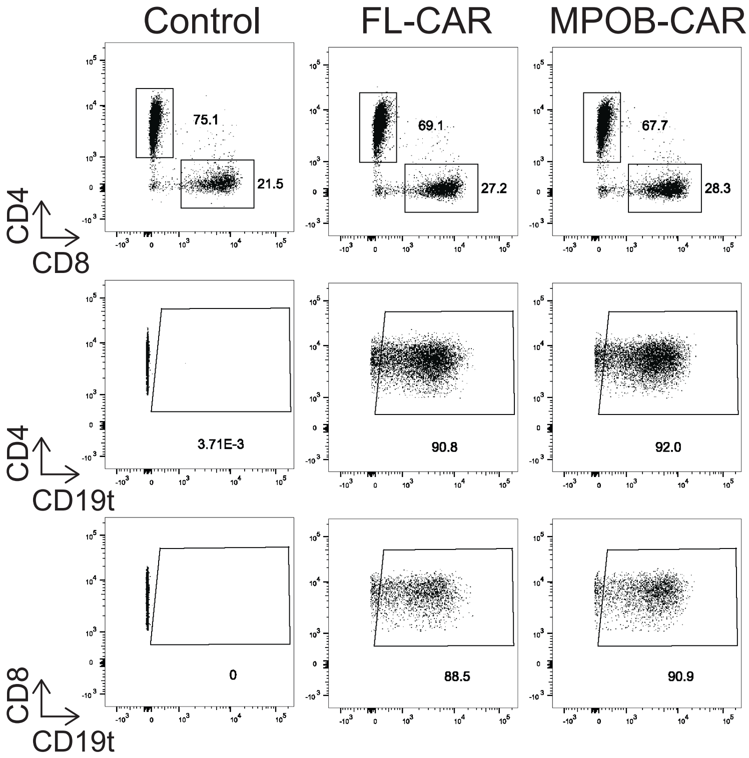

Figure S1. Expression of small molecule-specific-CARs in T cell

Expression of FL-CAR and MPOB-CAR in primary human T cells.

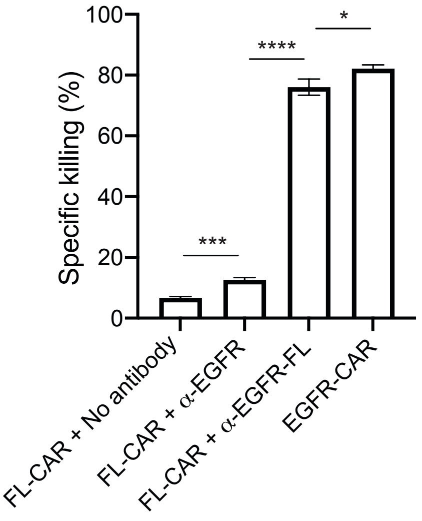

Figure S2. Small molecule-specific CAR is as efficient as conventional CAR in tumor cell killing

Cytotoxicity of FL-CAR and conventional EGFR-CAR T cells were compared. MDA-BM-468 cells were labeled with α-EGFR or α-EGFR-FL and were co-cultured with FL-CAR or EGFR-CAR T cells (n=3). *P* values were determined by unpaired Student's *t* test. *$P < 0.05$, ***$P < 0.001$, ****$P < 0.0001$.

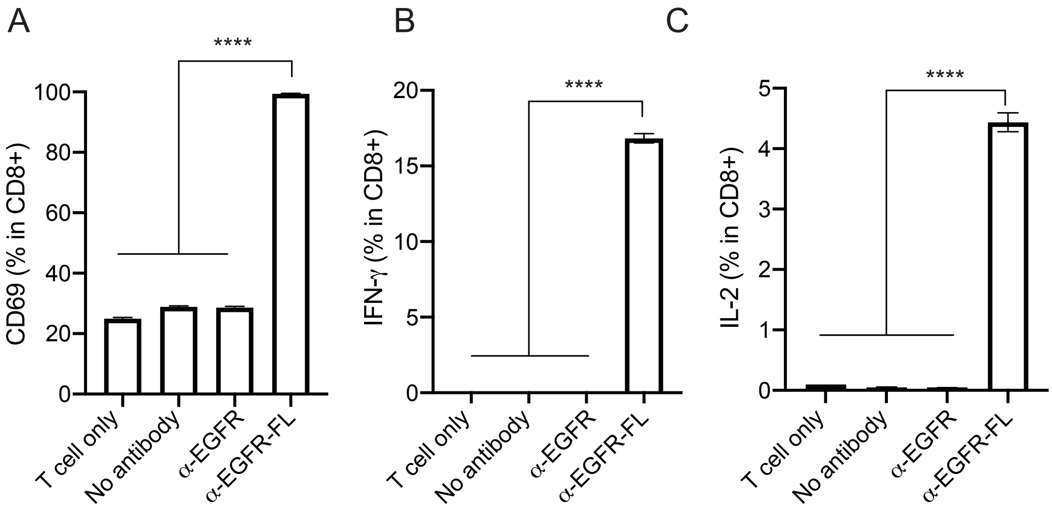

Figure S3. Small molecule-specific activation of CAR T cell

(A-C) Expression level of (A) CD69, (B) IFN-γ and (C) IL-2 in FL-CAR T cells were measured after co-cultured with or without MDA-MB-468 cells labeled with α-EGFR or α-EGFR-FL (n=3). *P* values were determined by unpaired Student's *t* test. ****$P < 0.0001$.

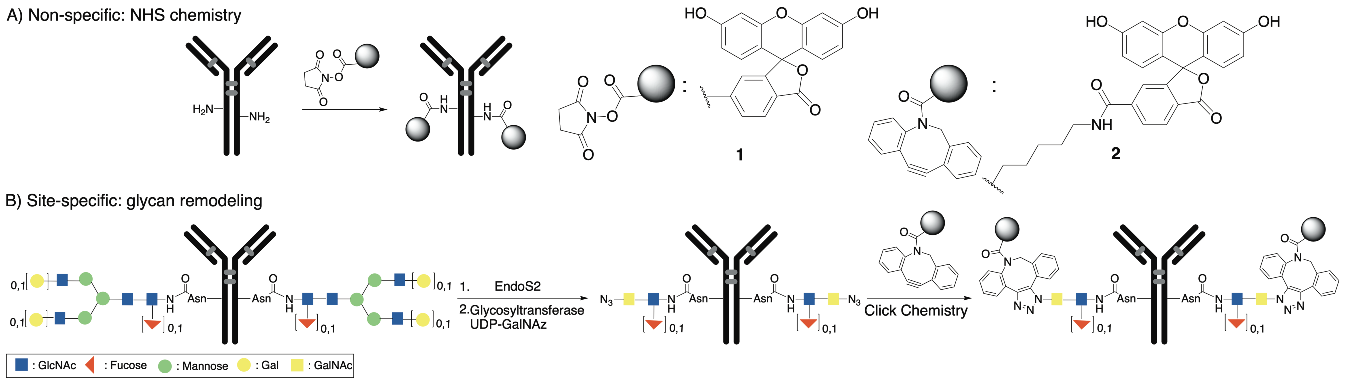

Figure S4. Conjugation of fluorescein to antibody

(A) Non-specific chemical acylation of amines (i.e., lysine and N-terminus) on the antibody. (B) Site-specific conjugation of asparagine 297 on the antibody via glycan remodeling and strain-promoted azide-alkyne cycloaddition (SPAAC) click chemistry.

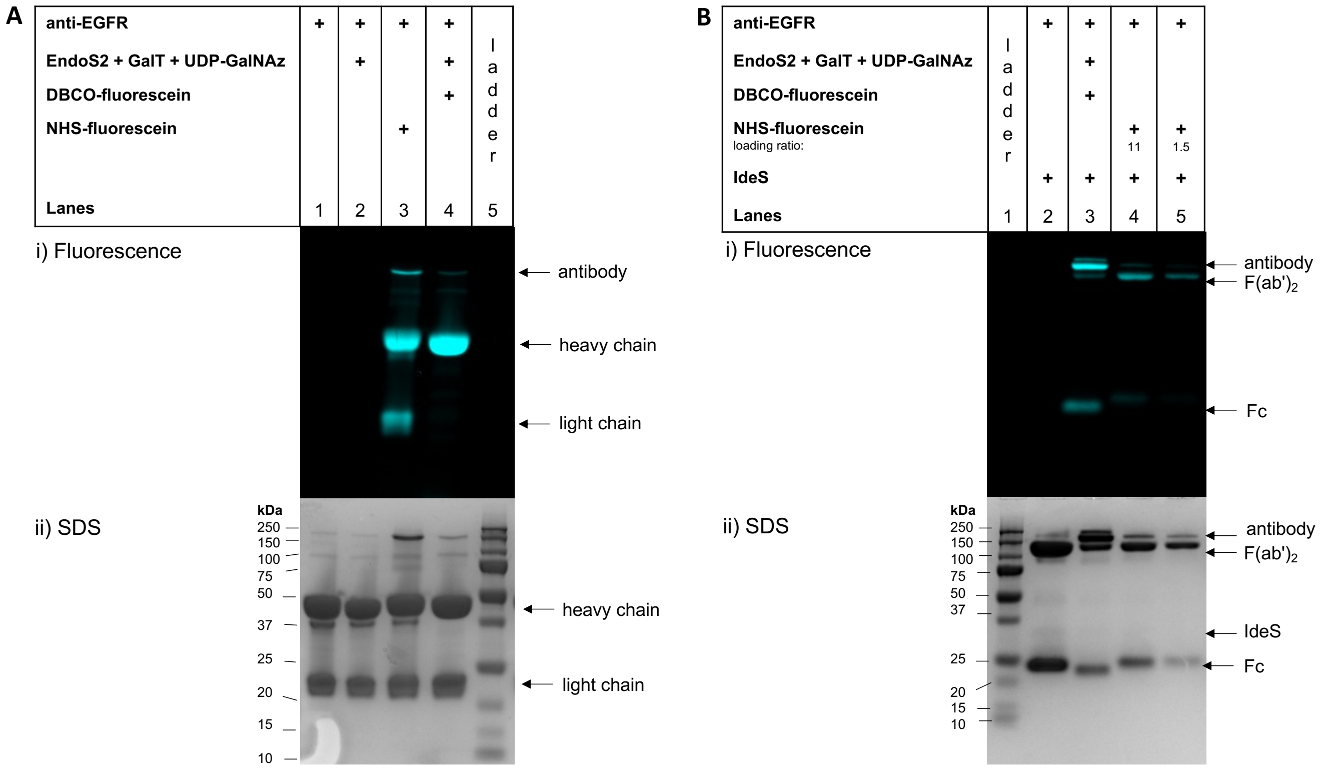

Figure S5. Analysis of antibody-fluorescein conjugates constructed via glycan remodeling (site-specific) or amine-reactive NHS (non-specific) chemistries.

(A) Characterization of the antibody-chromophore conjugates by reducing SDS-PAGE. Lanes: (1) anti-EGFR antibody; (2) anti-EGFR antibody treated with endoglycosidase (EndoS2), and subsequently treated with galactosyltransferase (GalT) and UDP-N-azidoacetylgalactosamine (UDP-GalNAz); (3) anti-EGFR antibody incubated with fluorescein-NHS ester (NHS-FL); (4) cetuximab treated with endoglycosidase (EndoS2), subsequently treated with GalT and UDP-GalNAz, and lastly incubated by dibenzocyclooctyne-fluorescein (DBCO-FL); (5) Molecular weight standards. (B) Characterization of the antibody-chromophore conjugates by non-reducing SDS-PAGE. Lanes: (1) Molecular weight standards; (2) cetuximab treated with IdeS; (3) cetuximab treated with endoglycosidase (EndoS2), subsequently treated with GalT and UDP-GalNAz, and lastly incubated by dibenzocyclooctyne-fluorescein (DBCO-FL); (4) and (5) cetuximab incubated with fluorescein-NHS ester (NHS-FL). The gels were assessed via fluorescence imaging (excitation 455–485 nm and emission 510–555 nm, top) and Coomassie staining (bottom).

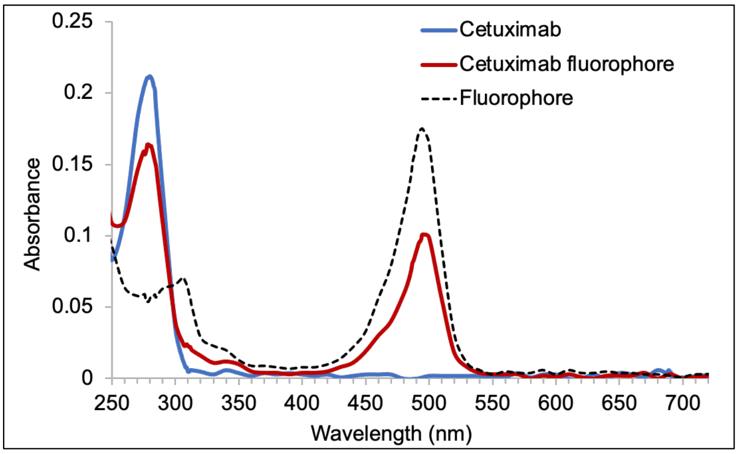

Figure S6. Representative UV-vis spectra of anti-EGFR antibody-fluorescein conjugates constructed via glycan remodeling (site-specific).

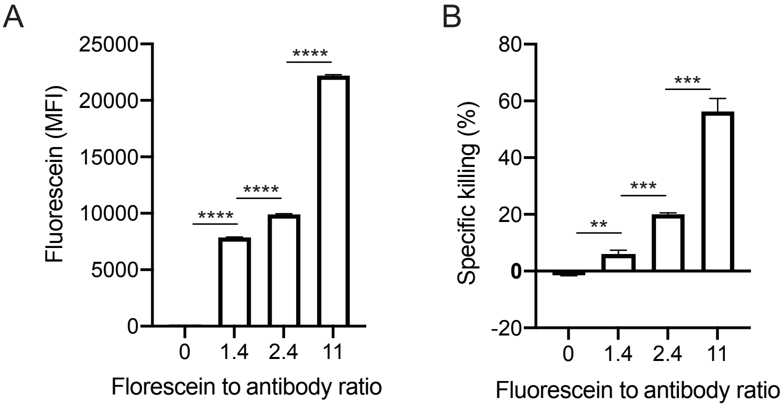

Figure S7. Small molecule-dose and density-dependent activation of CAR T cell

(A) anti-EGFR antibody was conjugated to fluorescein at different ratio (fluorescein to antibody ratio=0, 1.4, 2.4 and 11). MDA-MB-468 cells were labeled with these conjugates at antibody concentration of 10nM, and the fluorescence intensity was measured by flow cytometry (n=3). (B) Cytotoxicity of FL-CAR T cells were tested after co-culture with these labeled MDA-MB-468 cells (n=3). *P* values were determined by unpaired Student's *t* test. $^{**}P < 0.01$, $^{***}P < 0.001$, $^{****}P < 0.0001$.

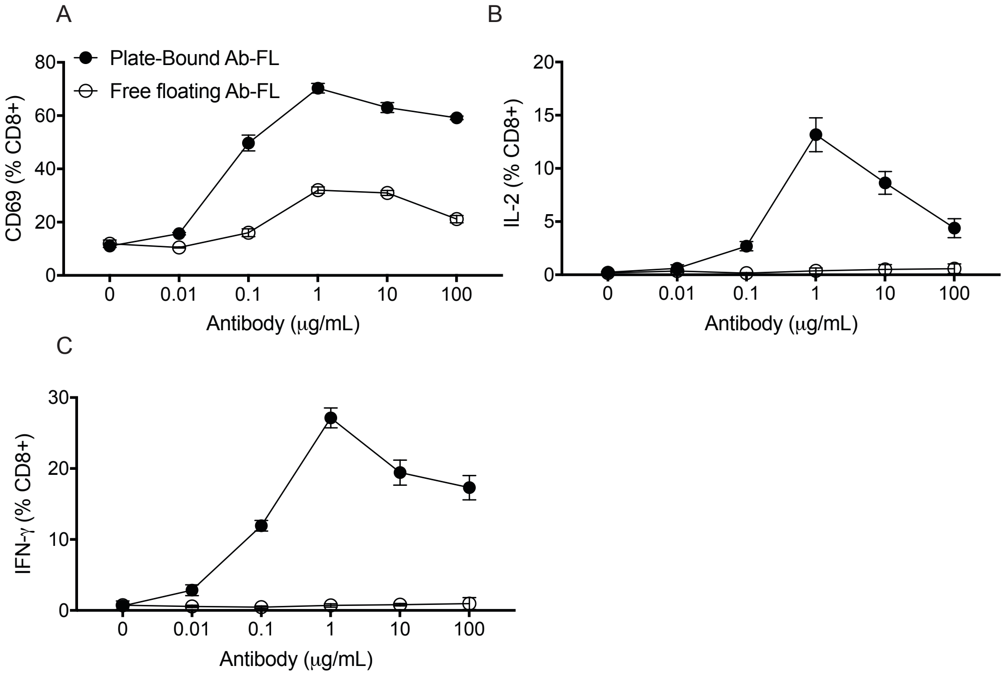

Figure S8. Small molecule-dose-dependent activation of CAR T cell

FL-CAR T cells were cultured on the plate coated with different concentrations of antibody-fluorescein conjugates (Ab-FL) (filled circle) or cultured with free floating Ab-FL (open circle) for 28 hrs, and expression level of (A) CD69, (B) IL-2 and (C) IFN-γ in FL-CAR T cells were measured.

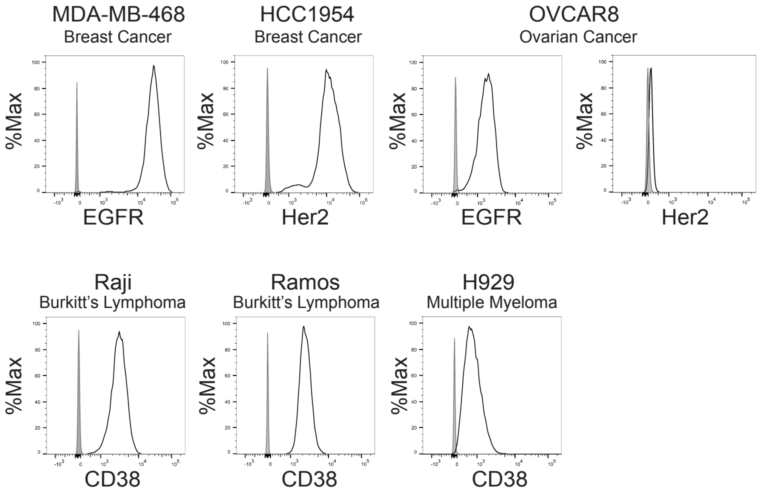

Figure S9. Expression of tumor antigens on cancer cell lines

Cell surface expression of tumor antigens on various cancer cell lines. Breast cancer: MDA-MB-468 (EGFR+) and HCC1954 (HER2+), ovarian cancer: OVCAR8 (HER2+ and EGFR+), Burkitt lymphoma: Raji (CD38+) and Ramos (CD38+), and multiple myeloma: H929 (CD38+).

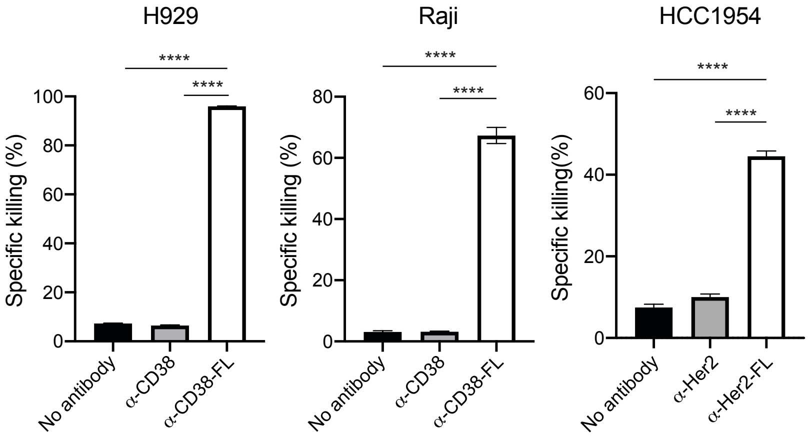

Figure S10. Redirectable small molecule specific-CAR system against various types of targets and tumors

Cancer cell lines were labeled with naked or fluorescein-conjugated antibodies specific to each target, and cytotoxicity of FL-CAR T cells were tested after co-culture with these labeled cancer cell lines (n=3). *P* values were determined by unpaired Student's *t* test. ****$P < 0.0001$.

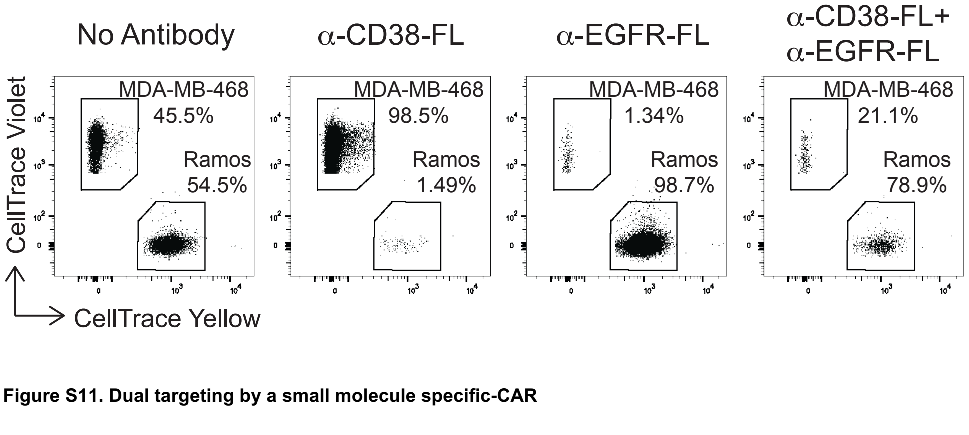

Figure S11. Dual targeting by a small molecule specific-CAR

Representative flow cytometry plots and of MDA-MB-468 cells (CellTracer Violet+) and Ramos cells (CellTracer Yellow+) survived after co-culture with FL-CAR T cells.

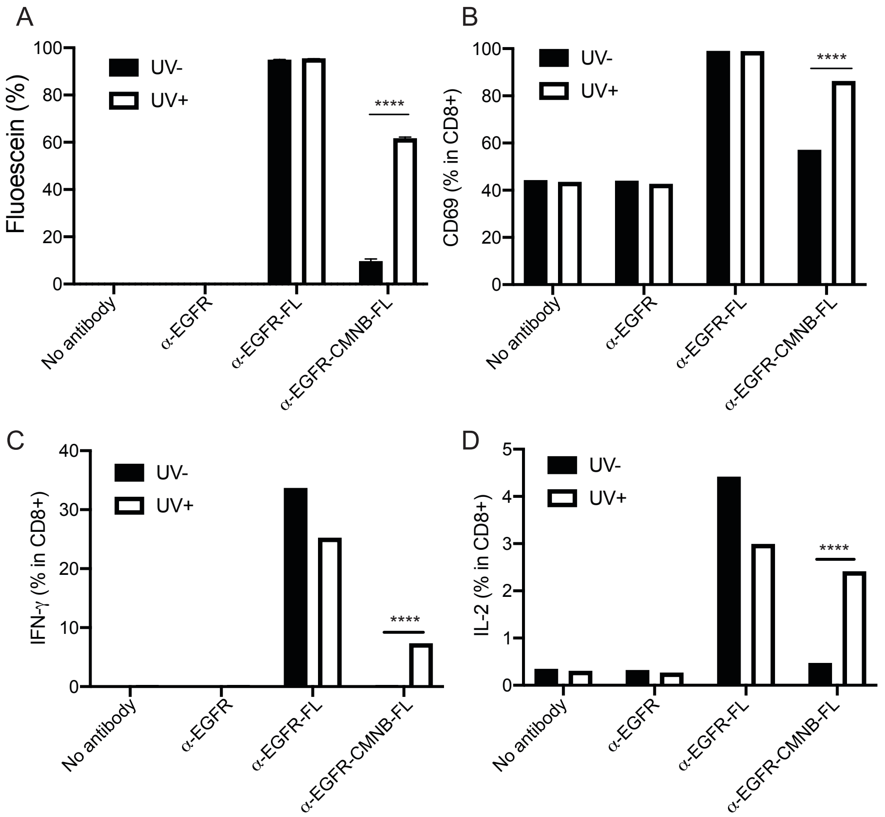

Figure S12. UV exposure-inducible activation of CAR T cells

(A) MDA-MB-468 cells were labeled with α-EGFR, α-EGFR-FL or α-EGFR-CMNB-FL, treated with or without UV-light for 10min, and the fluorescence intensity was measured (n=3). (B-D) Expression level of (B) CD69, (C) IFN-γ, and (D) IL-2 in FL-CAR T cells were measured after co-culture with MDA-MB-468 cells treated as in (A) (n=3). *P* values were determined by unpaired Student's *t* test. *****P* < 0.0001.

3. Supplementary Table

**Antibody**	**anti-EGFR antibody**		
**Chemistry**	Non-specific: NHS chemistry		Site-specific: GlyClick
**Chromophore (Ex/Em (nm))**	Fluorescein (494/520)		
**Antibody-chromophore ratio**	1.44 ± 0.01	11.18 ± 0.15	2.36 ± 0.08

Table S1. Antibody conjugates' chromophores loading ratio.

4. Author Contributions

A.K. and A.N. designed and performed experiments and interpreted data. C.D.N., R.D., A.N. and S.C.N. conceived light-sensitive caging system and designed experiments. A.S. and Z.S.Z. designed and performed experiments conjugating fluorescein to antibodies. A.K. and C.D.N. wrote the manuscript. The manuscript was revised and approved by all authors. C.D.N supervised the project and interpreted data.

5. Supplementary References

[1] K. S. Midelfort, H. H. Hernandez, S. M. Lippow, B. Tidor, C. L. Drennan, K. D. Wittrup, *J Mol Biol* **2004**, *343*, 685-701.
[2] S. Li, K. R. Schmitz, P. D. Jeffrey, J. J. Wiltzius, P. Kussie, K. M. Ferguson, *Cancer Cell* **2005**, *7*, 301-311.
[3] H. Büeler, R. C. Mulligan, *Mol Med* **1996**, *2*, 545-555.
[4] A. Sadiki, E. M. Kercher, H. Lu, R. T. Lang, B. Q. Spring, Z. S. Zhou, *Photochem Photobiol* **2020**, *96*, 596-603.
